# Lipid peroxidation is essential for α‐synuclein‐induced cell death

**DOI:** 10.1111/jnc.13024

**Published:** 2015-03-01

**Authors:** Plamena R. Angelova, Mathew H. Horrocks, David Klenerman, Sonia Gandhi, Andrey Y. Abramov, Mikhail S. Shchepinov

**Affiliations:** ^1^ UCL Institute of NeurologyQueen SquareLondonUK; ^2^ Department of ChemistryUniversity of CambridgeCambridgeUK; ^3^ Retrotope, Inc.Los Altos HillsCaliforniaUSA

**Keywords:** deuterated PUFA, lipid peroxidation, oxidative stress, α‐synuclein

## Abstract

Parkinson's disease is the second most common neurodegenerative disease and its pathogenesis is closely associated with oxidative stress. Deposition of aggregated α‐synuclein (α‐Syn) occurs in familial and sporadic forms of Parkinson's disease. Here, we studied the effect of oligomeric α‐Syn on one of the major markers of oxidative stress, lipid peroxidation, in primary co‐cultures of neurons and astrocytes. We found that oligomeric but not monomeric α‐Syn significantly increases the rate of production of reactive oxygen species, subsequently inducing lipid peroxidation in both neurons and astrocytes. Pre‐incubation of cells with isotope‐reinforced polyunsaturated fatty acids (D‐PUFAs) completely prevented the effect of oligomeric α‐Syn on lipid peroxidation. Inhibition of lipid peroxidation with D‐PUFAs further protected cells from cell death induced by oligomeric α‐Syn. Thus, lipid peroxidation induced by misfolding of α‐Syn may play an important role in the cellular mechanism of neuronal cell loss in Parkinson's disease.

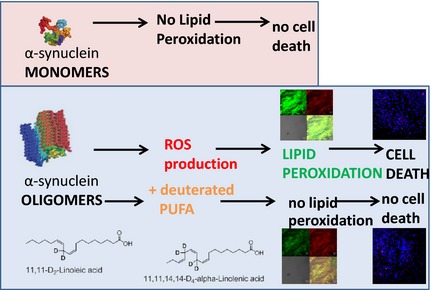

We have found that aggregated α‐synuclein‐induced production of reactive oxygen species (ROS) that subsequently stimulates lipid peroxidation and cell death in neurons and astrocytes. Specific inhibition of lipid peroxidation by incubation with reinforced polyunsaturated fatty acids (D‐PUFAs) completely prevented the effect of α‐synuclein on lipid peroxidation and cell death.

**Abbreviations used**
HEt: dihydroethidium
Lin: linoleic acid
Lnn: linolenic acid
PD: Parkinson's Disease
PI: propidium Iodide
PUFA: polyunsaturated fatty acids
ROS: reactive oxygen species
α‐Syn: α‐synuclein

Although Parkinson's disease is a complex multifactorial disorder, one key causal factor remains the misfolding and aggregation of the protein α‐Syn. The major histopathological hallmarks of Parkinson's Disease (PD) include the loss of dopaminergic neurons in substantia nigra and the presence of Lewy bodies, which are intracellular inclusions of aggregated α‐Syn. The exact mechanism by which aggregation of α‐Syn induces neuronal cell death in the course of the disease is not yet clear; however, a growing body of evidence points towards a key role of oxidative stress in PD pathogenesis (Gandhi and Abramov 2012).

Reactive oxygen species (ROS) and even mild lipid peroxidation have been shown to play important roles in physiological signal transduction (Vaarmann *et al*. 2010; Domijan *et al*. 2014), but overproduction of ROS may lead to oxidative damage to DNA, proteins and/or to lipid membranes. The extent of tissue damage through oxidation depends on the tissue composition and on the ability of the intracellular antioxidant system to restore ROS production to basal levels. The brain is particularly prone to oxidative damage, due to the high level of oxidation‐prone polyunsaturated fatty acids (PUFAs), high rates of ROS production due to high oxygen consumption and energy turnover and low levels of endogenous antioxidants (Halliwell 2006). Twenty per cent of all energy generated by the body is utilized by the brain, of which a striking 25% (i.e. the 5% of the total energy generated) is spent on maintaining and repairing oxidatively damaged lipid membranes (Brenna and Carlson 2014).

We have previously shown (Cremades *et al*. 2012) that exposure of neurons and astrocytes from a mixed primary culture to oligomeric forms of α‐Syn leads to a dramatic increase in the basal ROS production.

In this study, we investigate how α‐Syn‐induced ROS production may contribute to cell death by generating lipid peroxidation. We applied low concentrations of recombinant monomeric or oligomeric α‐Syn to primary co‐cultures and measured ROS production as well as lipid peroxidation. Furthermore, we modulated the lipid peroxidation using exogenously applied PUFAs in order to ascertain the relevance of lipid peroxidation on cell toxicity.

CNS tissues are rich in polyunsaturated fatty acids (PUFA) (Alessandri *et al*. 2004) which can be built enzymatically from two essential PUFAs, linoleic acid (C18:2,n‐6) and α‐linolenic acid (C18:3,n‐3) (Brenner 1974). PUFAs are highly prone to a non‐enzymatic chain reaction of autoxidation (Yin *et al*. 2011). Initiated by ROS, this process can damage multiple PUFA residues within lipid membranes. The success of antioxidant approaches to mitigate Parkinsonism and inhibit associated lipid peroxidation has been limited (Halliwell 2011). An alternative method (Shchepinov 2007) employs deuteration at the bis‐allylic sites (Scheme 1) to slow down the rate‐limiting step of hydrogen abstraction, resulting in strong inhibition of the chain reaction of lipid peroxidation (Hill *et al*. 2012). It has been successfully tested in several lipid peroxidation‐related neurological disease models including PD (Shchepinov *et al*. 2011) and Friedreich's ataxia (Cotticelli *et al*. 2013). Here, we employ lipid peroxidation‐ resistant D‐PUFAs to obtain further mechanistic insights into α‐Syn pathophysiology and ways to prevent it.

**Scheme 1:**
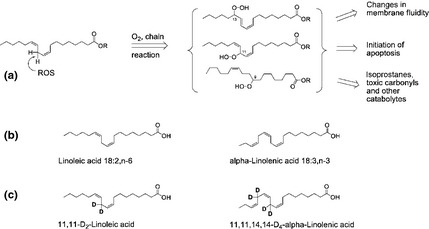
(a) Chain reaction of lipid peroxidation is initiated by a reactive oxygen species (ROS)‐mediated hydrogen abstraction from a bis‐allylic site within a polyunsaturated fatty acids (PUFA). The carbon‐based radical thus formed reacts with O_2_ to yield an alkylperoxy radical, which abstracts hydrogen from a neighbouring PUFA in the membrane. This propagation of the chain process can be terminated by antioxidants or radical recombination. The chain process generates a large variety of toxic moieties including peroxides and carbonyls. (b) The essential PUFAs, n‐6 Lin and n‐3 Lnn, from which higher essential PUFAs can be built *in vivo* by enzymatic desaturation and extension. (c) Deuteration at the bis‐allylic sites protects D2‐Lin and D4‐Lnn from the rate‐limiting step of lipid peroxidation.

## Materials and methods

### Materials

#### Deuteration of PUFAs

Deuterated Poly‐Unsaturated Fatty Acids (D‐PUFAs) 11, 11‐D2‐linoleic acid and 11,11,14,14‐D4‐α‐linolenic acid were prepared as described previously (Hill *et al*. 2011) and used as free acids. Non‐deuterated PUFAs were obtained from Sigma–Aldrich (99%; St. Louis, MO, USA). Cells were pre‐incubated with 10 μM H‐ or D‐PUFA in the culturing media for 24 h prior to experiment and washed with HBSS before experiments.

#### Aggregation of monomeric α‐Syn

##### Generation and characterization of monomeric and oligomeric α‐synuclein

The A90C mutant variant of α‐Syn was purified as a monomeric fraction from Escherichia coli and labeled with either maleimide‐modified Alexa Fluor 488 (AF 488), Alexa Fluor 594 (AF 594) or Alexa Fluor 647 (AF647) dyes (Invitrogen, Carlsbad, CA, USA) via the cysteine thiol moiety. We utilized the method described in Cremades et al. 2012; to aggregate fluorescently labeled α‐Syn monomers 50% labeled with AF488 [fluorescence resonance energy transfer (FRET) donor], and 50% with AF647 or AF594 (FRET acceptors). For the aggregation reactions, equimolecular concentrations of the AF488‐ and AF647‐labeled A90C α‐Syn in Tris 25 mM (pH 7.4) and 0.1 M NaCl (with 0.01% NaN_3_ to prevent bacterial growth during the experiments) were mixed to give a final volume of 300 μL, bringing the total protein concentration to 1 mg/mL (70 μM). The solutions were incubated in the dark at 37°C, with constant agitation at 200 rpm for 4–8 days, during which time aliquots were taken. At each time point, a 2 μL aliquot was diluted 10^5^‐fold by serial dilution with 0.022 μm‐filtered buffer (Tris 25 mM [pH 7.4] and 0.1 M NaCl) for smFRET analysis at 25°C. As oligomers form, they are statistically likely to contain both a donor and acceptor label, and FRET is able to occur between these, allowing them to be separated from the vast majority of monomeric protein making up the sample. For this study we stopped the aggregation at 29 h, and single‐molecule confocal FRET analysis confirms the presence of ~ 0.8% oligomers (number of detected oligomers as a fraction of total number of detected events), 82% of which exhibit a higher FRET efficiency characteristic of the compact, beta‐sheet oligomers previously identified (Cremades et al. 2012), the other 18% having a lower FRET efficiency.

### Methods

#### Primary cultures

Mixed cultures of cortical neurons were prepared as described previously (Suwanjang *et al*. 2013) with modifications, from male and female Sprague–Dawley rat pups 2–4 days post‐partum (UCL breeding colony). Experimental procedures were performed in full compliance with the United Kingdom Animal (Scientific Procedures) Act of 1986.

Rat brains were quickly removed and macerated in chilled Ca^2+^‐free HBSS (Invitrogen). The tissue was minced and trypsinized (0.1% for 15 min at 37°C), triturated and plated on poly‐d‐lysine‐coated coverslips and cultured in Neurobasal A medium (Gibco‐Invitrogen, Paisley, UK) supplemented with B‐27 (Gibco‐Invitrogen), 2 mM l‐glutamine and 1% Penicillin/Streptomycin. Cultures were maintained at 37°C in a humidified atmosphere of 5% CO_2_ and 95% air, media changed twice a week. Neurons were easily distinguishable from glia: they appeared phase bright, had small smooth rounded somata and distinct processes, and lay just above the focal plane of the glial layer.

#### Imaging of superoxide generation and lipid peroxidation

Superoxide generation was measured with Dihydroethidium (HEt; 2 μM, Invitrogen). Culturing media was washed off with HBSS and all imaging was performed in HBSS (Gibco). To avoid accumulation of oxidized products, HEt was not preloaded to the cells, but was added to the solutions in the beginning of the experiments in primary co‐cultures. Phototoxicity and photobleaching of cells were minimized by limiting the light exposure to the time of acquisition of the images. Fluorescent images were acquired with a frame interval of 10s. Data were analysed using software from Andor IQ (Belfast, UK).

The rate of lipid peroxidation was measured using confocal microscopy. Confocal images were obtained with a Zeiss 710 Laser Scanning Microscope (LSM) with an integrated Meta detection system. To assess lipid peroxidation, C11‐BODIPY (581/591, 2 μM, Molecular Probes) was excited using the 488 and 565 nm laser and fluorescence measured from 505 to 550 nm and above 580 nm (40× objective). For measurements, BODIPY 655/675 (2 μM, Molecular Probes) was excited by 563 and 630 nm lasers and measured from 580 to 610 and above 650 nm (40× objective). DAF‐FM was measured using excitation 488 nm and emission 510–560 nm. Illumination intensity was kept to a minimum (at 0.1–0.2% of laser output) to avoid phototoxicity and the pinhole set to give an optical slice of ~ 2 μm. Addition of a bright field image allowed separation between neurons and glia, that are visibly different and are situated on different focal planes. Data were acquired and analysed using ZEN2009 software.

#### Toxicity experiments

For toxicity assays cells were exposed to 5 μM propidium iodide (PI) and 5 μM Hoechst 33342 (Molecular Probes, Eugene, OR) for 30 min prior to imaging. The PI is excluded from viable cells and exhibits a red fluorescence following a loss of membrane integrity, while the Hoechst 33342 labels all nuclei blue. This allows expression of the number of dead (red stained) cells as a fraction of the total number of nuclei counted (blue stain). Using phase contrast optics, a bright field image allowed identification of neurons, which look quite different to the flatter glial cells and also lie in a different focal plane, above the glial layer. A total number of 100–300 neurons were counted in 4–5 fields of each coverslip. Each experiment was repeated four or more times using separate cultures.

### Statistical and data analysis

Statistical analysis and data analysis were performed using Origin 9 (Microcal Software Inc., Northampton, MA, USA) software. Results are expressed as means ± standard error of the mean (SEM).

## Results

### The ability of alpha‐synuclein to produce ROS depends on the state of aggregation

**Figure 1:**
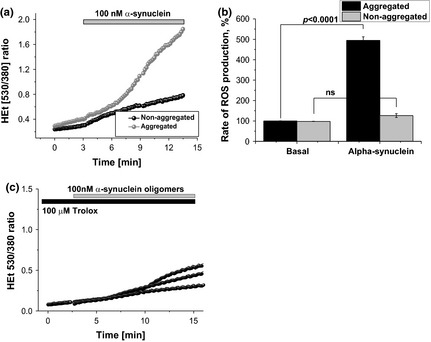
The ability of α‐synuclein to induce reactive oxygen species (ROS) is dependent on the form of its conformation. ROS production is significantly higher when primary glio‐neuronal co‐culture was treated with aggregated (oligomeric) form of alpha‐synuclein, compared to those treated with non‐aggregated (monomeric) form of alpha‐synuclein (a and b). (a) shows representative traces of dihydroethidium (HEt) measurements whereas (b) summarizes the effects of the two forms of alpha‐synuclein on the ROS production in percentage. ns: not significant. C – effect of pre‐incubation (20 min) of neurons and astrocytes with 100 μM Trolox on α‐synuclein‐induced ROS production.

In agreement with our previous publication (Cremades *et al*. 2012) we have found that in rat cortical primary co‐cultures of neurons and astrocytes, application of 100 nM aggregated a‐Syn (consisting of ~ 99 nM monomeric a‐Syn and ~ 1 nM oligomeric a‐Syn) but not non‐aggregated (100 nM monomeric) α‐Syn significantly increased the rate of ROS production (Fig. 1a), measured as the rate of increase in HEt ratio (494.6 ± 17.6%, *n* = 9, *p* < 0.0001; and 126.3 ± 19.5%, *n* = 9, *p* = 0.1962; of basal rate 100%, *n* = 9; accordingly, Fig. 1b). Thus, aggregated but not the non‐aggregated α‐Syn induces ROS formation, indicating that only the aggregated form exerts a substantial influence on the cellular ROS production.

This α‐Syn‐induced ROS production can be significantly reduced by pre‐incubation of the cells (for 20 min) with water soluble analogue of vitamin E – trolox (100 μM; Fig. 1c; *n* = 8 experiments).

### Lipid peroxidation is a part of the alpha‐synuclein‐induced damage to neurons

**Figure 2:**
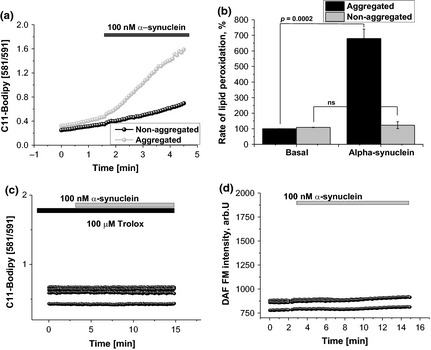
Aggregated, but not non‐aggregated form of α‐synuclein produces lipid peroxidation. Equivalent amounts of aggregated and non‐aggregated α‐synuclein have been tested for their ability to evoke lipid peroxidation in mixed glio‐neuronal culture, using the C‐11 BODIPY 581/591 lipid peroxidation probe. The addition of the aggregated form of α‐synuclein only, induced lipid peroxidation. (a), representative traces showing the increase in basal rate of lipid peroxidation only after addition of oligomeric α‐synuclein, (b), summary of the results given in percentage and (c), effect of 100 μM Trolox on lipid peroxidation under exposure of aggregated α‐synuclein. (d) Effect of aggregated α‐synuclein on DAF‐FM fluorescence.

Normally, production of ROS can be quenched by an effective antioxidant system which maintains redox balance. However, in certain conditions ROS overproduction takes place, which cannot be balanced by the cellular antioxidant system and this leads to oxidative stress. Application of 100 nM aggregated α‐Syn (equivalent of 99 nM monomeric and 1 nM oligomeric a‐Syn) significantly increases the rate of lipid peroxidation in primary cultures from the cortex (679.5 ± 60.9% of basal rate, *n* = 125; *p* < 0.001 Fig. 2a and b), measured as the rate of C11‐BODIPY 581/591 ratio. The effect of monomeric 100 nM a‐Syn was indistinguishable from the basal rate (123.17 ± 22.1% of basal rate; *n* = 132 cells; Fig. 2a and b). α‐Syn penetrates the cells in 5–8 min (Cremades *et al*. 2012) that suggests that this peptide can induce lipid peroxidation in all – plasmalemmal and intracellular membranes. Lipid peroxidation was more likely to be induced by α‐Syn‐stimulated ROS production because pre‐incubation with 100 μM Trolox, can also completely block α‐synuclein‐induced increase in C11‐BODIPY 581/591 ratio (Fig. 2c). To avoid any possible interaction of C11‐BODIPY 581/591 ratio with peroxynitrite, we measured the effect of aggregated α‐Syn on NO production in the co‐culture of neurons and astrocytes. Using DAF‐FM as an indicator for NO, we have found that aggregated α‐Syn has no effect on NO production at the same time with the activation of lipid peroxidation (Fig. 2d).

**Figure 3:**
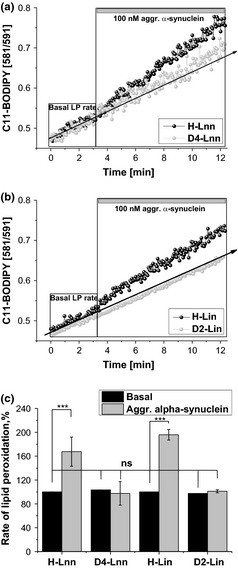
α‐synuclein‐induced lipid peroxidation could be prevented by deuterated polyunsaturated fatty acids (PUFAs). No changes in the basal rate of lipid peroxidation (representative traces) are evoked by aggregated α‐synuclein in D4‐Lnn (a and c) and D2‐Lin (b and c) – pre‐treated co‐cultures from rat neurons and astrocytes in C‐11 Bodipy assay in comparison to the non‐deuterated forms of PUFA (H‐Lin and H‐Lnn). (c), Histogram summarizing changes in the rate of lipid peroxidation (in percentage) in the presence of deuterated PUFAs. ***p < 0.0001; n.s. non‐significant.

**Figure 4:**
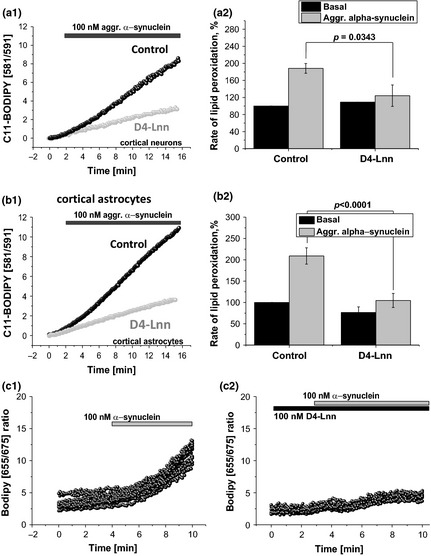
Deuterated polyunsaturated fatty acids (PUFAs) prevent α‐synuclein‐induced rise in lipid peroxidation in both, cortical neurons and cortical astrocytes. Aggregated form of α‐Syn stimulates lipid peroxidation in both neurons (a1, a2) and astrocytes (b1, b2), however, statistically significant level of the protective D4‐Lnn effect is reached only in astrocytes (*p* < 0.0001). c1, c2 – experiments, using alternative lipid peroxidation indicator BODIPY 655/675.

To test if D‐PUFAs can block α‐Syn‐induced lipid peroxidation we treated cortical neurons and astrocytes with D‐PUFA (both D4‐Lnn and D2‐Lin) and normal H‐PUFA controls (Lnn and Lin acids). We have found that even control PUFAs reduced the effect of oligomeric α‐Syn on lipid peroxidation, perhaps due to substituting higher, more oxidation‐prone PUFAs present in membranes. Thus, in the presence of PUFAs, the α‐Syn‐mediated increase in the rate of C11‐BODIPY 581/591 ratio in neurons and astrocytes from co‐cultures was only 188.3 ± 11.5%, compared to 679.5 ± 60.9% when D‐PUFAs were absent, *n* = 9, Fig. 3a). A total of 24‐h pre‐incubation of the cells with D‐PUFAs significantly reduced the rate of the lipid peroxidation in mixed primary cultures treated with 100 nM aggregated α‐Syn to basal (to 104.7 ± 16.5% from basal rate, *n* = 9 experiments, *p* = 0.0005; Fig. 3b). We have found no differences in the effects of D4‐Lnn and D2‐Lin on the rate of lipid peroxidation (Fig. 3a and c). It should be noted that the effect of α‐Syn was independent of the cell type when comparing with astrocytes (209.1 ± 19.2%, *n* = 9 experiments) and neurons (188.3 ± 11.5%, *n* = 9 experiments; Fig. 4a and b). The data, obtained using C11‐BODIPY 581/591 were confirmed by another indicator for lipid peroxidation BODIPY 655/675 (Fig. 4c). Thus, oligomeric but not monomeric α‐Syn induces a significant increase in lipid peroxidation which can be blocked partially by pre‐treatment of the cells with H‐PUFAs and completely by pre‐treatment of the cells with D‐PUFAs.

### Isotope‐reinforced PUFAs protect against alpha‐synuclein‐induced cell death

**Figure 5:**
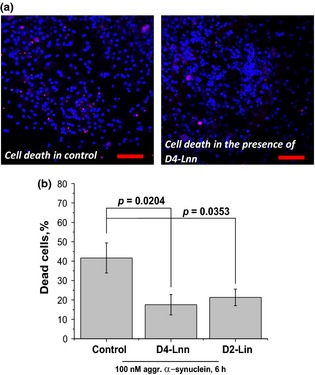
Protective effect of deuterated polyunsaturated fatty acid (PUFA) against α‐synuclein‐induced cell death. About 100 nM aggregated (but not non‐aggregated) alpha‐synuclein significantly increased the number of dead cells in primary glio‐neuronal cultures (a). Pre‐incubation of the cells with deuterated PUFA‐containing medium (either D4‐Lnn or D2‐Lin) significantly reduced the number of dead cells (given in percent) in the culture (a and b). Cell death was assessed using PI/Hoechst assay to label dead cells and total number of cells, respectively.

In our experiments, 6‐h exposure of cortical co‐culture of neurons and astrocytes to 100 nM aggregated α‐Syn (99 nM monomeric a‐Syn, 1 nM oligomeric a‐Syn) results in a significant increase in the number of dead cells (to 41.7 ± 7.8%, *n* = 9 experiments, Fig. 5a and b). To investigate if D‐PUFAs have a protective effect on the survival of neurons and astrocytes upon prolonged exposure to aggregated α‐Syn, we assessed the number of Hoechst/PI‐positive cells in a classical cell death assay. About 24‐h pre‐incubation of the cells with either an n‐6 (D2‐Lin) or an n‐3 (D4‐Lnn) PUFA significantly protects the cells against α‐Syn‐induced cell death (17.5 ± 5.2%, *n* = 9, *p* = 0.0204 and 21.3 ± 4.3%, *n* = 9, *p* = 0.0353, when compared to cell death evoked by aggregated α‐Syn alone – 41.7 ± 7.8%, *n* = 9; Fig. 5b). These experiments strongly suggest that lipid peroxidation is an important part of the mechanism of α‐Syn ‐induced neuronal cell death that occurs in Parkinson's disease, and that this effect can be mitigated by D‐PUFAs.

## Discussion

It has been previously demonstrated that α‐Syn aggregates to generate a toxic beta‐sheet oligomeric structure that generates high levels of ROS (Cremades *et al*. 2012). While it is recognized that overproduction of ROS results in oxidation of a range of molecules including DNA, protein and lipids (Gandhi and Abramov 2012), our study provides definitive evidence for the induction of lipid peroxidation by oligomeric α‐Syn. Importantly, we are also able to demonstrate that the application of D‐PUFAs prevents α‐Syn‐induced lipid peroxidation as well as α‐Syn‐induced cell death. Since D‐PUFAs possess the unique ability to prevent lipid peroxidation, with no other non‐specific free radical scavenging or antioxidant properties, this confirms that lipid peroxidation induced by α‐Syn is indeed the major mechanism of toxicity of cells exposed to oligomers.

There is a homology between segments of α‐Syn and fatty acid‐binding proteins, suggesting that α‐Syn can function as a lipid‐binding protein (Perrin *et al*. 2001). Interaction of α‐Syn with PUFAs, particularly with neuron‐specific higher PUFAs such as docosahexanoic acid, affects its oligomerization and further aggregation (De *et al*. 2011), resulting in increased cytotoxicity. α‐Syn aggregates interact with vesicular transport, and can embed themselves into lipid membranes forming pores, and affecting membrane permeability (Fecchio *et al*. 2013). Also, α‐Syn can bind to membranes, fatty acids and intracellular lipid droplets as reviewed in Ruiperez *et al*. 2010;. But rather than a fatty acid carrier, it is the binding to membranes that seems to be the intrinsic property of α‐Syn (Lucke *et al*. 2006). Interestingly, α‐Syn oligomers are formed upon interaction with peroxidation‐prone PUFAs but not with monounsaturated fatty acids, while non‐saturated fatty acids actually reduce the level of oligomerization (Sharon *et al*. 2003). Given this and the fact that binding of lipid peroxidation‐promoting transition metals such as iron or copper exacerbates oligomerization of α‐Syn (Amer *et al*. 2006), we suggest that α‐Syn may also have a specific affinity for peroxidized lipids. Thus, monomeric α‐Syn may increase resistance to apoptosis, while α‐Syn oligomers have the opposite effect (Ruiperez *et al*. 2010). While lipid peroxidation features prominently in both apoptosis and α‐Syn‐associated pathology, the detailed pro‐ and anti‐apoptotic properties of α‐Syn and its oligomeric forms, as well as their complex interactions with PUFAs in mitochondria await further elucidation (Ruiperez *et al*. 2010).

Mitochondrial lipid peroxidation has been linked to PD (Dexter *et al*. 1986) and apoptosis, which itself may be linked to several lipid peroxidation events through different possible pathways, including a recently described ‘lipid whiskers’ mechanism (Greenberg *et al*. 2008). Cytochrome c has to be released into the cytosol from the inner mitochondrial membrane to trigger apoptotic caspase cascades. For this to happen, cardiolipin, a major PUFA‐rich building block of inner mitochondrial membranes, has to be oxidized (Kagan *et al*. 2005). This oxidation can be triggered by elevation of ROS production in the vicinity of PUFA‐rich mitochondrial membranes. Other apoptosis‐initiating steps may include the loss of membrane potential (Zamzami *et al*. 1995) as a result of lipid peroxidation‐driven change in the membrane fluidity (Dobretsov *et al*. 1977). Membrane‐bound peroxidized PUFAs decompose into numerous toxic moieties. Reactive carbonyls such as 4‐hydroxynonenal (HNE), 4‐hydroxy‐2‐hexenal (HHE), malondialdehyde (MDA), acrolein and many other can damage proteins, lipids and nucleic acids and activate the caspase cascades (Ellis 2007). Despite the fact that it was shown that monomers could bind to fatty acids and form oligomers upon reacting with products of lipid peroxidation (Broersen *et al*. 2006), in our hands only exogenously applied oligomers could produce ROS, thus inducing lipid peroxidation.

The observed pernicious role of the ROS‐induced chain reaction of lipid peroxidation in the toxicity of aggregated a‐Syn suggests that down‐regulating lipid peroxidation may have a mitigating effect on PD progression. However, attempts to use antioxidants to inhibit the ROS‐inflicted damage in lipid peroxidation and elsewhere have so far been unsuccessful (Halliwell 2011). This may be due to a variety of factors, including (a) near‐saturating levels of antioxidants present normally, (b) preventing important signalling function played by ROS, (c) antioxidants switching to pro‐oxidant mode under certain conditions, (d) challenges of delivering the antioxidants to the particular cellular locations where they are most required and (e) the need to constantly supply antioxidants as they are used up in the process of ROS quenching. An alternative strategy, that of making PUFAs more resistant to lipid peroxidation through site‐specific deuteration, is free of these drawbacks. Hydrogen abstraction off a bis‐allylic site, the rate‐limiting step of the process, can be slowed down by deuteration due to the kinetic isotope effect. The kinetic isotope effect is further multiplied through the chain format, essentially resulting in the inhibition of lipid peroxidation, with stochiometric (non‐chain) enzymatic reactions of lipid oxidation affected to a much lesser degree. The essential nature of PUFAs would ensure efficient delivery of reinforced PUFAs to the sites with elevated lipid peroxidation, making D‐PUFAs a clinically relevant therapeutic approach for mitigating PD.
